# Editorial: biological ontologies and semantic biology

**DOI:** 10.3389/fgene.2014.00018

**Published:** 2014-02-04

**Authors:** John M. Hancock

**Affiliations:** Department of Physiology, Development and Neuroscience, University of CambridgeCambridge, UK

**Keywords:** semantic biology, biological ontologies, semantic web, data representation, data analysis

As the amount of biological data and its diversity accumulates massively there is a critical need to facilitate the integration of this data to allow new and unexpected conclusions to be drawn from it.

The Semantic Web comprises web-based technologies that allow linking of data between diverse data sets. Semantic Biology is the application of semantic web technology in the biological domain (including medical and health informatics). The Special Topic in Biological Ontologies and Semantic Biology brings together papers in this broad area—which spans computer science, computational biology and bioinformatics—providing a platform for strengthening what is still a new and underappreciated area of research.

A key aspect of semantic biology is the description of biological, and biology-related, entities using ontologies. Ontologies are a critical requirement for such integration as they allow conclusions drawn about biological experiments, or descriptions of biological entities, to be understandable and integratable despite being contained in different databases and analyzed by different software systems. Ontologies are the standard structures used in biology, and more broadly in computer science, to hold standard terminologies for particular domains of knowledge. They consist of sets of standard terms, which are defined and may have synonyms for ease of searching and to accommodate different usages by different communities. These terms are linked by standard relationships, such as “is_a” (an eye “is_a” sense organ) or “part_of” (an eye is “part_of” a head). In this way more detailed (granular) terms can be linked to broader terms, allowing computation to be carried out that takes these relationships into account.

The classical biological ontology is the Gene Ontology (GO) (Ashburner et al., 2000) which addresses aspects of gene function, the processes in which they participate and the localization of gene products. Increasingly, semantic biology requires the linkage of these concepts to other biological features. Three such biological entities are included in the Special Topic. The Anatomical Entity Ontology (AEO) (Bard, 2012) provides a typology of anatomical entities across species that is linked to cell types (via links to the cell ontology). Amongst others things, this allows linkage of anatomical structures across species, allowing inferences of homology and comparison of features such as gene and protein expression across species.

Another cross-species ontology, and one that complements work on anatomy, is described by Giudicelli and Lefranc (2012). They provide an update on the IMGT-Ontology which is an ontology of immunogenetics and immunoinformatics used in the international ImMunoGeneTics information system® (http://www.imgt.org). The IMGT-Ontology describes a range of immunogenetics concepts (immunoglobulins or antibodies, T cell receptors, major histocompatibility (MH) proteins of humans and other vertebrates, proteins of the immunoglobulin superfamily and MH superfamily, related proteins of the immune system of vertebrates and invertebrates, therapeutic monoclonal antibodies, fusion proteins for immune applications, and composite proteins for clinical applications).

A key problem for semantic biology is linking data on phenotypic measurements between model organisms, used to understand human disease, and clinical observations made in humans. This has been an active area of research in recent years (Hancock et al., 2009; Schofield et al., 2010). Shimoyama et al. (2012) make an important contribution to this area by describing a set of ontologies used to describe clinical measurements, measurement methods and experimental conditions for traits common to rat and man (and, by extension, in other mammalian model systems such as mouse and, potentially, more distantly related species). These measurements are similar to those used in large-scale phenotyping experiments (Hancock and Gates, 2011) so that this ontology system provides a potentially valuable mechanism for the study of genotype-phenotype relations in mammals.

Going beyond the underlying ontological structures used to describe biological data Imam et al. (2012) describe an integrated set of ontologies used within the Neuroscience Information Framework (www.neuinfo.org/), which describe major domains in neuroscience, including diseases, brain anatomy, cell types, sub-cellular anatomy, small molecules, techniques, and resource descriptors. This application provides a valuable insight into how sets of existing ontologies can be integrated with novel, more application-specific ontologies and structures to underpin a semantic-based knowledge system. NIF links logically consistent sets of terms into single structures but forms links between these logically consistent sets using bridging modules. Deb (2012) argues for an alternative approach using a single upper level (foundational) ontology to link specific biological domain ontologies.

A key issue that any such framework raises is how to compare and choose appropriate ontologies for any given system. A typical default position in biological applications is to accept the ontologies held in the open biological ontologies set (Smith et al., 2007). Here Klie and Nikoloski (2012) argue that ontology choice is to a degree application-specific and that domain-specific ontologies may in some cases be more useful than general ontologies such as the GO.

The major purpose of developing biological ontologies (rather than simpler controlled vocabularies) is to make use of the relations implicit in ontologies to facilitate analysis and annotation. These topics are addressed by two papers in this series. Ross et al. (2013) describe the use of the PRotein Ontology to carry out cross-species comparisons of function in the spindle checkpoint pathway. Bastos et al. (2013) consider the use of subsets of functionally coherent proteins to improve functional annotation in a protein family.

Finally, advances in technology provide new opportunities for the use of semantically-enriched data in applications that are only minimally ontology-aware. Dönitz and Wingender (2012) describe a web-based service that can be accessed from any application to make use of standard ontologies, removing a significant burden to application development. At a higher level, Deb and Srirama (2013) provide us with a view of how the data and ontologies currently being produced might be linked and accessed via cloud infrastructures and describe some of the problems this raises in the domain of human eHealth.
